# Social Network Supported Process Recommender System

**DOI:** 10.1155/2014/349065

**Published:** 2014-01-15

**Authors:** Yanming Ye, Jianwei Yin, Yueshen Xu

**Affiliations:** ^1^College of Computer Science and Technology, Zhejiang University, Hangzhou 310027, China; ^2^College of Information Engineering, Hangzhou Dianzi University, Hangzhou 310018, China

## Abstract

Process recommendation technologies have gained more and more attention in the field of intelligent business process modeling to assist the process modeling. However, most of the existing technologies only use the process structure analysis and do not take the social features of processes into account, while the process modeling is complex and comprehensive in most situations. This paper studies the feasibility of social network research technologies on process recommendation and builds a social network system of processes based on the features similarities. Then, three process matching degree measurements are presented and the system implementation is discussed subsequently. Finally, experimental evaluations and future works are introduced.

## 1. Introduction

Workflow technology has gained further application and development with the rapid growth of modern business environment. And process models are being widely used in the development of organizational structures [1], information systems [2], service-oriented architectures [3], and web services [4]. However, business process modeling is either complex or time-consuming, which often involves in selecting concrete activities to be performed, determining their execution order, dealing with the exceptions that may occur, and so forth. Besides, in modern commerce, both frequent changes of custom demands and the specialization of the business process necessitate the ability of modeling business processes in an effective and efficient way for enterprises. Thus, many business intelligence (BI) based techniques have been adopted to improve the business process modeling work, such as process mining and process retrieval [5–7]. Both process mining and process retrieval are complex or need more manual works. For improving the efficiency, some process modeling technologies so-called business process recommendation are proposed recently. However, most of the existing process recommendation technologies are only based on the static structure analysis of the process and the other properties of a process, such as the performer behaviors and the editor intensions, are not considered. In practice, when creating a new process, a modeler is inclined to refer to the modeling conduct of the familiar users or the processes with the same modeling intension or the process fragments that are used more frequently by certain users. This paper presents a social network supported method that can recommend the processes or process parts using the social features of process modeling, such as intension, activity performers, usage frequency, and modeling history.

This paper is organized as follows. After this introduction and the related works in Section 2, Section 3 gives some definitions with related basic instructions, and at the end of this section how to calculate process matching degree is highlighted. In Section 4, we discuss the implementation of the social network based process recommender system. In Section 5, some experiments on the system are discussed. Finally, Section 6 is devoted to research perspective.

## 2. Related Work

A process model is often in form of some graphical notations and describes how a certain process is composed out of different tasks, in which resources are involved in carrying out these tasks and objects are manipulated [8, 9]. Therefore, most of the existing process mining methods recur to graph minings, especially graphic structure mining. As for the process recommendation technologies in process modeling, most of the methods are described as how to find certain process fragments as a subgraph that is familiar with the given process part. In [10], a workflow recommendation technique called FlowRecommender was proposed, which was announced that it can leverage provenance of workflows to provide recommendation for the best node that needs to be chosen to complete the workflow. Paper [11] used a NMSF (near neighbor and maximal subgraph first) process matching method to recommend the next nodes for the reference process fragment.

Social network has been widely researched in the last decade and various social network systems have been built, some of which also produce great commercial success. Social network analysis (SNA) has become an important branch of scientific studies. The related studies cover a wide range of different fields, including sociology, psychology, economy, and computer science [12]. SNA can effectively mine the implied information in social networks. Therefore, if it can use the SNA methods, the process modeling will be more effective. At first, we must construct a social network from a process repository. As far as we know, only a very small quantity of researches [12, 13] are directly related. In [12], three types of social networks can be generated from a process repository and a recommendation system, including (1) social network from process models, (2) social network from user history, and (3) social network from insertion history. Unfortunately, the three social networks are constructed for only having the network structure but more social features of the network cannot be used in the system. For example, the social network (1) is described to be more like an overlap of a processes graph, of which the nodes refer to the performers in the process models and the arcs show the transfer of work between the performers. In the social network (2), the authors build a matrix of users and processes in which each cell contains the number of uses of a process model by a user then calculate the distance between any two users by several measures, such as *Minkowski distance, Hamming distance, *and* Pearson's correlation coefficient*. By setting a certain threshold value, unimportant arcs could be removed and the left directly connected users are seen in different cliques, respectively. But these distance measures employ Euclidean distance or do not consider for the process correlation. Moreover, there are no other social features that can be used in this social network (2). The social network (3) is built on the relationship of process creators and is more suitable for change propagation process than process modeling.

From the discussion above, the greatest contribution of paper [12] is to show that the modeling support system can be extended with capabilities to take social information into account. But the three social networks that paper [12] proposed are insufficient in the SNA. This paper presents a method to build a unique social network that involves in more social features.

## 3. Preliminaries

Business process is a series of activities that are performed by different performers to specific targets, respectively, and the order of activities represents collaboration of these performers. In workflow systems, the process reflects the actual business process, and the activity node represents business operation in enterprise. Information or operations will flow or be conducted in turn according to the nodes sequence (or by the arcs). Therefore, the business process model can be abstracted as a directed graph, of which the nodes stand for activities and the arcs stand for orders.

### 3.1. Social Network from Business Process

**Definition 1 (business process model)**

Let (*P*, *T*, *F*) be a WF-net (as defined in [14]), and a process model, PM, is a 5-tuple (*P*, *T*, *F*, *U*, Ψ) where

- *R*  is a set of performers,
- Ψ : *T* → *R* is the function of performer (*U*) assignment for a task (*T*).

In a workflow net, *P*, *T*, and *F* refer to places, transitions, and flow relations, respectively. The models in the process repository are represented in terms of a Petri net, in which activities are modeled by transitions, and casual dependencies are modeled by places and arcs, while performers related to activities are specified in the above transitions.

Next, we give a formal definition of social network as the following.

**Definition 2 (social network model)**

A social network, SN, is a graph denoted by a 5-tuple SN = (*U*, *A*, *E*, *R*, Φ), where

- *U* is the finite set of nodes and each node stands for a user,
- *A* is a set of attributes of users,
- *E*⊆*U* × *U* is the finite set of edges,
- *R* = {*R*_1_,…, *R*_*m*_} is a finite set of relationships,
- Φ : *U* × *U* → *R* is the relationship function.

As the main manifestation of social network, SNS (social network software/social network site) has gained huge success in business and become the most concerned topic in related research fields. In [15], SNS is defined as follows:

*…web-based services that allow individuals to (1) construct a public or semi-public profile within a bounded system, (2) articulate a list of other users with whom they share a connection, and (3) view and traverse their list of connections and those made by others within the system.*

A more recent definition is proposed by Kwon and Wen [16] who define SNS as “*websites that allow building relationships online between persons by means of collecting useful information and sharing it with people. Also, they can create groups which allow interacting amongst users with similar interests.”*

SNS is more and more applied or studied in business domain and also attracts research attention as marketing instruments [17–22], while the globalization and virtualization of enterprise business make efficient business process management (BPM) increasingly important. This inspires us to study how to apply SNS and the related researches in BPM. Note that the activities in business process are modeled by transitions and the transitions and places are both defined as nodes in WF-net. At the same time, performers related to activities are specified in the transitions. Therefore, the performers can be specified as users in the SNS and the order by which performers execute the tasks can be specified as the friend relationship in the SNS.

**Figure 1:**
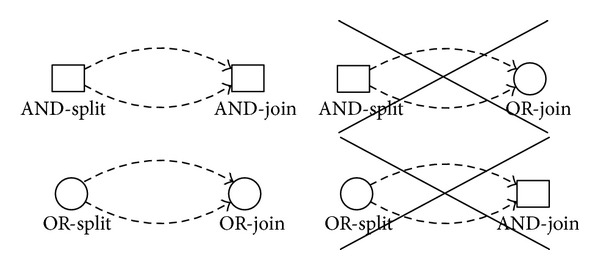
Good and bad constructions.

Almost in all cases, a process has four main structures, including AND-split, OR-split, and AND-join, OR-join. In short, the type “*AND*” means enforcement and the type “*OR*” means “select” or “condition execution.” Intuitively, the combination of the *split* and *join* will be a total of four kinds of forms. However, a “good” workflow can balance AND/OR-splits and AND/OR-joins [23]; that is, two parallel flows initiated by an AND-split should not be joined by an OR-join. Two alternative flows created via an OR-split should not be synchronized by an AND-join. As shown in Figure 1, an AND-split should be complemented by an AND-join and an OR-split should be complemented by an OR-join [23]. So, in fact, there are only two kinds of forms (shown in the left of Figure 1) that own well structures. The other two kinds of forms (shown in the right of Figure 1) are defined as deadlocks and lack of synchronization, respectively. And various methods can be used to detect these structure errors [24–26].

Given that every process is verified not to have bad constructions before it is added into the process repository, it is enough to consider the good constructions only. Therefore, we can only consider the AND-split or OR-split and the AND-join or OR-join will be implied in the good constructions.

For simplicity's sake, the places and transitions can be combined as nodes, and the tasks, performers, split type, join type, and other properties of places and transitions can be added into the nodes' properties. Under this idea, business process is a series of activities that is performed by different performers to specific targets, respectively, and the order of activities represents collaboration of these performers. In a workflow system, the process reflects the actual business process, and the activity node represents business operation in enterprises. Information or operations will flow or be conducted in turn according to the nodes sequence. Therefore, the business process model can be abstracted as the directed graph, of which the nodes stand for activities, with a corresponding label (implicating activity type, content, the serial number, etc.), and the edges stand for orders.

**Definition 3 (business process graph)**

Let *T* be a set of node types and let *L* be a finite alphabet of labels for nodes. A business process is a connected graph denoted by a 6-tuple *P* = (*N*, *E*, *α*, *β*, *s*, *e*), where

- *N* is the finite set of nodes,
- *E*⊆*N* × *N* is the finite set of edges,
- *α* : *N* → *T* is the node typing function,
- *β* : *N* → *L* is the node labeling function,
- *s* ∈ *N* is the start node,
- *e* ∈ *N* is the end node.

Node *x* ∈ *N* is the input of node *y* ∈ *N* if and only if there exists a directed edge connecting *x* to *y* (i.e., (*x*, *y*) ∈ *E*). Node *x* ∈ *N* is the output of node *y* ∈ *N* if and only if there exists a directed edge connecting *y* to *x* (i.e., (*y*, *x*) ∈ *E*). The node that has no input node is called *start* node and the node that has no output node is called *end* node. In a workflow repository, any business process can be modeled as a process graph having only one *start* node and only one *end* node. So the business process graph *P* in Definition 3 can also be simplified as *P* = (*N*, *E*, *α*, *β*).

However, in a social network site, for example, twitter or Sina Weibo, there exist several interexchange methods, such as tweet, @, reply, and retweet. Tweet is the conduct of publishing a message, retweet is to forward a message that others tweeted without any change, @ is used to push the message to others by force, and reply tends to accept message self-selectively with no or more additional information. Thus, when every performer in a social network site is a user, their relationships that are implied in process structure can be regarded as information exchange in the social network. For example, if there is a node “*A*” with AND-split type connecting to nodes “*B*” and “*C*” in a process, the performer of “*A*” will be a user “*User_A”* in corresponding social network who replies a message that is received from upper user (that is performer of parent node) with the additional information about the node label “label_*A*” firstly. Then @ the message to users “*User_B*” and “*User_C*” who will reply the message in response. Similarly, if there is a node “*A*” with OR-split type connecting to nodes “*B*” and “*C*” in a process, the performer of “*A*” will be a user “*User_A”* in corresponding social network who only replies a message that is received from upper user with the additional information about the node label, and then the users “*User_B”* and “*User_C”* will reply the message. Such a proceeding makes the generated social network retain most socialization properties, and the study on message interaction can be used to analyse the dynamic features of business process. Compared to (1), social network from process models are more about static features based on graph algorithms that are used in network research.

It is important to note that the users being performers in the same process only depend on whether they @ or reply the same topic (the message series) when reconstructing a process from social network. But what is the first message in a message series? We define a message series code for each message series and the code is also the only representative of the response process. So the message series code can also be called as process code. The first message in a message series is the series code that is published by the process creator. So a whole process can be found from its creator in the social network. In actual applications, a user can search for suitable process models and generate a new model by combining them; for example, user “U1” created a process P1 and tweeted the process code C1, and user “U2” created a process P2 and tweeted the process code C2. When user “U3” creates a new process P3, he may use the P1 and P2 as parts of the P3. Then in the social network, “U3” must retweet C1 and C2 in turn before he tweets the process code C3 and the C3 must contain the C1 and C2. Next, the performer of the first node of P3 will reply the message C3,…, and so forth. Thus, study on social network (2) from user history can be transformed to relationship mining from users that @ or reply the same or related topic. Likewise, study on social network (3) from insertion history can be transformed to relationship mining from users that retweet related topics.

Finally, three types of social networks defined in [12] can be replaced by the only social network constructed by the method that this paper presents. And the social network retains as much social features as possible. However, how to define message series code as the sole representative of a process is a problem that has to be solved. To facilitate reading, the message series code will be called as process code in the following, and the definition will be given.

### 3.2. Process Code

DFS (depth first search) and BFS (breadth first search) are widely used in graph mining algorithms. Compared with BFS, DFS consumes less memory but runs slower due to the stack utilization. On the contrary, the BFS uses more memory but runs faster than DFS. The facts in construction practice of process repositories and process patterns show that the vast majority of business processes are simply structural and with less nodes (in process repository used in this paper, there are 86% processes containing less than 20 nodes, and of which nearly 71% processes contain less than 10). Thus, the problem of memory usage is no longer an issue. On the contrary, because the number of processes may be huge, the time performance is more important. Therefore, this paper uses BFS to define the process code.

Breadth first search by different node orders of the same hierarchy may lead to different BFS sequences. Therefore, this paper presents the standard BFS sequence to ensure that the BFS sequence of the same process is unique. And we can reconstruct unique process with the standard BFS sequence. Next, we give the definitions about BFS code based on BFS sequence.

**Definition 4 (BFS code)**

The breadth first traversal order on a process graph *P* is a linear order. As Definition 3 shows that the directed edge of *P* can be labeled by ordered nodes that the edge is connected to; therefore, BFS code of *P* can be represented as the following:

(1) BFSsequence=s#{sni:sni∈E}#{ninj:ninj∈E}#⋯{nte:nte∈E}#e,

where the symbol # is the separator that divides the different traversal hierarchies.

**Figure 2:**
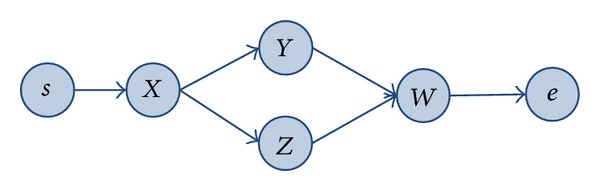
Process sample *P*.

For example, as shown in Figure 2, BFS code of the process sample *P* is

(2) $$\begin{array}{c} \text{BFSsequence}\left(P\right)=s\#sX\#XY,\;XZ\#YW,\;ZW\#We\#e\text{.} \end{array}$$

**Definition 5 (standard BFS code)**

The BFS code of process *P* is called standard BFS code if the labels of the same hierarchies are by lexicographic order denoted as BFSsequence(*P*).

Except for (2), the BFS code of process that is shown in Figure 1 can also be *s*#*sX*#*XZ*, *XY*#*ZW*, *YW*#*We*#*e*, or others. But only (2) follows the Definition 5, so only (2) can be called standard BFS code of the process.

Next, we give the definition of process node label that is used in the process code.

**Definition 6 (process node label)**

A process node label is a value of label function *l* : *F* ∪ *E* ∪ *C* → *Ω*, where

- *F* is a finite set of functions;
- *E* is a finite set of events;
- *C* is a finite set of connectors, and that is {and, or};
- *Ω* is a set of text labels.

The process code is composed of each node's label, while the message that a user replies or @ comprises the relevant functions, events, and connector.

### 3.3. Process Matching Degree

Note that if a process is a fragment of the other process, then the first process matches the other, but the opposite is not always true. However, for simplicity, we do not consider the difference and regard them matching with each other.

In different intention, process matching degree is related to user matching degree, structural matching degree, and behavioral matching degree.

User matching degree between two processes can be measured based on their creators, performers, and reusers, and thus the user matching degree can be divided into three parts: creator matching degree, performer matching degree, and reuser matching degree. Let UC be a set of creators of a process and the processes that it reuses, and |UC| is the length of the UC; then the creator matching degree between two processes *p*1 and *p*2 is

(3) $$\begin{array}{c} Mat\_creator\left(p1,p2\right)=\frac{\left|\text{UC}\left(p1\right)\cap \text{UC}\left(p2\right)\right|}{\min\left(\left|\text{UC}\left(p1\right)\right|,\left|\text{UC}\left(p2\right)\right|\right)}. \end{array}$$

Let UP be a set of performers of a process, and |UP| is the length of the UP; then the performer matching degree between two processes *p*1 and *p*2 is

(4) $$\begin{array}{c} Mat\_performer\left(p1,p2\right)=\frac{\left|\text{UP}\left(p1\right)\cap \text{UP}\left(p2\right)\right|}{\min\left(\left|\text{UP}\left(p1\right)\right|,\left|\text{UP}\left(p2\right)\right|\right)}. \end{array}$$

Let UR be a set of creators of processes that a certain process is reused, and UR is the length of the UR; then the reuser matching degree between two processes *p*1 and *p*2 is

(5) $$\begin{array}{c} Mat\_reuser\left(p1,p2\right)=\frac{\left|\text{UR}\left(p1\right)\cap \text{UR}\left(p2\right)\right|}{\min\left(\left|\text{UR}\left(p1\right)\right|,\left|\text{UR}\left(p2\right)\right|\right)}. \end{array}$$

The process user matching degree is a combined metric that can be represented as the following:

(6) Mat_user(p1,p2)=α·Mat_creator(p1,p2)+β·Mat_performer(p1,p2)+λ·Mat_reuser(p1,p2),

where *α*, *β*, and *λ* are the adjustment coefficients that are set to 0.2, 0.7, and 0.1, respectively, in this paper.

The process structural matching degree can be calculated according to process matching degree that is based on BFS code [27]. In this paper, we present a novel metric that takes the information propagation path into account. Firstly, we define the social network schema and metapath as follows.

**Definition 7 (social network schema)**

A social network schema from social network model is *S* = (*O*, *R*), where

- *O* is the finite set of roles and each user in the social network corresponds to a role;
- *R* = {and, or} is relationships between the roles that different users to.

**Definition 8 (Metapath)**

A metapath of information propagation is a path defined on the network schema *S* = (*O*, *R*) and is denoted in the form of $O_{1}\overset{R_{1}}{\to }O_{2}\cdots O_{n}\overset{R_{n}}{\to }O_{n+1}$.

For example, in the process sample *p* in Figure 2, if the users of node belong to roles of *staff*, *accountant*, *manager* and *treasurer,* respectively, and the split type of *X* is AND-split, then the metapath may be $staff\overset{and}{\to }manager\overset{and}{\to }treasurer$. Meanwhile, if the users belong to roles of *staff*, *manager*, *accountant*, and *treasurer*, respectively, and the split type of *X* is OR-split, then path $staff\overset{or}{\to }manager\overset{and}{\to }treasurer$ and path $staff\overset{or}{\to }accountant\overset{and}{\to }treasurer$ are the two metapaths. If the roles are labeled with unique symbol (as shown in Table 1) and the relationships *and* and *or* are labeled as *R*_1_ and *R*_2_, then the two metapaths can be represented as string *O*_1_ *R*_2_ *O*_2_ *R*_1_ *O*_4_ and *O*_1_ *R*_2_ *O*_3_ *R*_1_ *O*_4_.

As we discussed above, a process may have one or more metapaths in the social network. The matching degree of the two metapaths can be measured by the SED [28] of the labeled metapaths.

**Definition 9 (string edit distance (SED))**

Given two strings *x* and *y*, the string edit distance of *x* and *y*, denoted as SED(*x*, *y*), is the minimum number of insertions, deletions, and substitutions to transform *x* into *y*. And the matching degree between metapaths *mp*_1_ and *mp*_2_ (labeled as *l* *mp*_1_ and *l* *mp*_2_, resp.) is

(7) Mat_lmp(mp1,mp2)=1−SED(lmp1,lmp2)−abs(|lmp1|−|lmp2|)min(|lmp1|,|lmp2|),

where |*l* *mp*_1_| and |*l* *mp*_2_| are the length of *lmp*_*1*_ and *lmp*_*2*_, respectively.

Secondly, we give the definition of process structural matching degree.

**Definition 10 (structural matching degree)**

Given two processes *p*1 and *p*2 and that *p*1 has *m* metapaths (from *p*1_*mp*_1_ to *p*1_*mp*_*m*_) and *p*2 has *n* metapaths (from *p*2_*mp*_1_ to *p*2_*mp*_*n*_), if *m* > *n*, then the structural matching degree is

(8) Mat_structure(p1,p2)=∑i=1nmax(Mat_lmp(p1_mpi,p2_mpj)) ∣ j∈{1,…,m}min(m,n).

The behavioral matching degree mainly measures the dynamic features about functions and events. Generally, functions and events are named by the unified naming conventions (if not, it can use the ETL tool and semantic analysis to standardize the names). Given processes *p*1 and *p*2, if *p*1has nodes with functions of *p*1_*f*_1_, *p*1_*f*_2_, …, *p*1_*f*_*m*_ and events of *p*1_*e*_1_, *p*1_*e*_2_, …, *p*1_*e*_*m*_, and *p*2has nodes with functions of *p*2_*f*_1_, *p*2_*f*_2_, …, *p*2_*f*_*n*_ and events of *p*2_*e*_1_, *p*2_*e*_2_, …, *p*2_*e*_*n*_, then the process behavioral matching degree between *p*1 and *p*2 can be calculated by the following formula:

(9) Mat_behavioral(p1,p2)  =(∑i=1m∑j=1n(IsSame(p1fi,p2fj)        +IsSame(p1ei,p2ej)))   ×(2×min(m,n))−1,

where *IsSame* (·) is the function that whether two parameters are equal in semantic sense and it returns 1 if they are equal and 0 if not. For simplicity, the behavioral matching degree can only be measured by functions and the formula will be as the following:

(10) $$\begin{array}{c} Mat_{behavioral(p1,p2)}=\frac{\sum_{i=1}^{m}\sum_{j=1}^{n}IsSame\left(p1_{f_{i}},p2_{f_{j}}\right)}{\min\left(m,n\right)}. \end{array}$$

## 4. Implementation

Social network supported process recommender system turns to help process builders fitting processes to achieve a modeling intention with regard to the other builders' modeling intention and modeling behavior.

**Figure 3:**
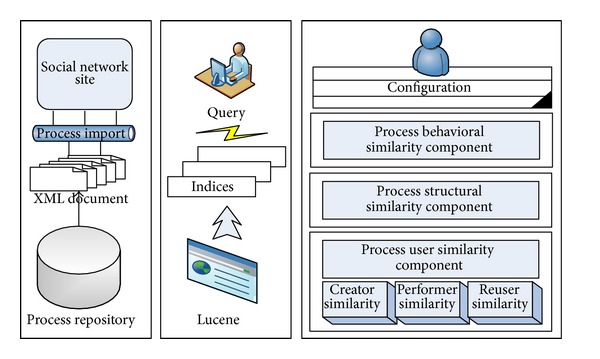
Structure of recommender prototype.

This recommendation system implementation consists of three parts (as shown in Figure 3) as follows.

1. A simple social network site meets our research needs by providing the basic functions of tweeting, retweeting, @ing, replying and searching, and so forth. A process import method is also provided in this part and each process, in the form of a well-formed XML document, can be added into the SNS database automatically.
2. A query interface allows users to request process models or process model parts that are of interest to them. In the system, we use open-source MySQL database to provide index capability, but not well in large dataset task. Therefore, we use Lucene to build indices on the objects of processes and network and provide query on creators, performers, reusers, functions, events, connectors, and so on through the query parser syntax.
3. A series of recommender components proposes appropriate process models which fit to a business process model that is currently being edited under different considerations. The parameters configuration is also provided for the users to determine the execution sequence of three types of process matching degrees and the adjustment coefficients for the process user matching degree. The thresholds of three matching degrees in certain recommending proceeding can be set through parameter configuration.

Different parameters values may lead to different efficiencies, even different results. In the next section, the difference will be demonstrated and discussed.

## 5. Empirical Evaluation

In this section, a comprehensive study is conducted in our experiments on synthetic dataset. The synthetic data generator that we use is similar to [10], and we also improve the generator with the ability to generate the processes with different kinds of structures (i.e., AND-split, OR-split, etc.). The experimental dataset contains 500 processes in total of which each activity is assigned to a performer and a function at random. And there totally contain 1000 virtual functions that can be semantically transformed to 200 different functions and 1000 virtual users that can be distinguished into 50 different roles.

**Figure 4:**
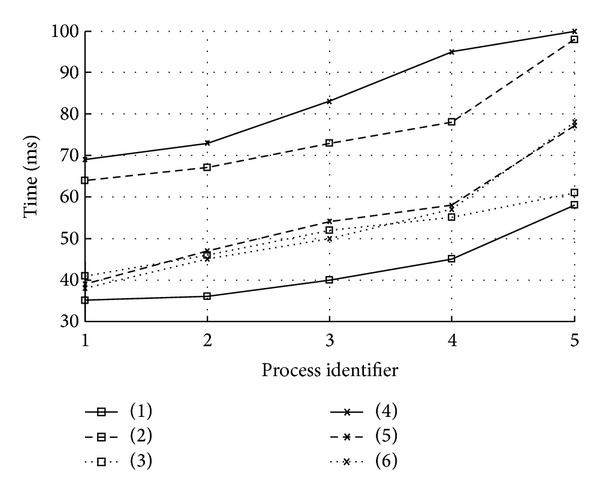
Experiment performance.

In the experiment, 5 process fragments (identified as (1), (2), (3), (4), and (5) in Figure 4) are extracted from the experimental dataset as the candidate processes and the three types of matching degrees between every candidate process and each process in experiment dataset are calculated under different thresholds groups (identified as (1), (2), (3), (4), (5), and (6) in Figure 4) in different orders successively. As it can be seen from Figure 4, the execution performance may vary considerably in different orders even under the same thresholds.

The average execution time and the average number of recommended processes are listed in Table 2.

In the same execution order, adjusting every threshold in turns may lead to big change in the results. From Table 2, the decrease of the threshold of structural matching degree makes the average execution time increase much more and this may be due to poor efficiency of the calculation of user matching degree and behavioral matching degree. Moreover, the decrease of the threshold of user matching degree makes the average number of recommended processes increase much more and this may be due to less overlapping between users of different processes in the process dataset.

## 6. Conclusion

We propose a social network supported process recommender and related techniques which can improve process modeling by providing reference processes from different perspectives to extend or complete the business process under construction, especially in some social considerations.

The similarities between process features and social network features are discussed in detail. And three types of process matching degrees between two processes are proposed by the consideration of contexts in different situations; then we implement the system prototype. The experimental evaluation shows that our method is efficient and effective for practical use.

The greatest contribution of this paper is that we show how to add social features to a recommendation-based process modeling support system. And the unique social network constructed from the process repository is first proposed which can assist the researchers in understanding social features in process modeling. The three types of process matching degrees are the initial attempt that benefits from the social network. At a more abstract level, this paper strengthens a research stream into process modeling that combines social software and social computing.

Still, much work has to be carried out in the future. For example, the user community with the similar interest may be more important for the modeling intention of process. Furthermore, process mining can benefit from the social network analysis. We hope that this paper can inspire more researchers to join in the newly ascendant field and many more efficient and effective methods or technologies can come to the fore.

## Figures and Tables

**Table 1:** Label of role.

Role	*Staff *	*Manager *	*Accountant *	*Treasurer *
Label	*O*_1_	*O*_2_	*O*_3_	*O*_4_

**Table 2:** Experiment result.

Parameters configuration		Average time (ms)	The average number of recommended processes
(1)	*θ*_*u*_ = 0.8	43	5
	*θ*_*s*_ = 0.8		
	*θ*_*b*_ = 0.8		
(2)	*θ*_*s*_ = 0.8	76	5
	*θ*_*b*_ = 0.8		
	*θ*_*u*_ = 0.8		
(3)	*θ*_*b*_ = 0.8	51	5
	*θ*_*u*_ = 0.8		
	*θ*_*s*_ = 0.8		
(4)	*θ*_*s*_ = 0.6	84	8
	*θ*_*b*_ = 0.8		
	*θ*_*u*_ = 0.8		
(5)	*θ*_*s*_ = 0.8	55	11
	*θ*_*b*_ = 0.6		
	*θ*_*u*_ = 0.8		
(6)	*θ*_*s*_ = 0.8	53	17
	*θ*_*b*_ = 0.8		
	*θ*_*u*_ = 0.6		
